# Insulin Receptor-Mediated Signaling via Phospholipase C-γ Regulates Growth and Differentiation in Drosophila

**DOI:** 10.1371/journal.pone.0028067

**Published:** 2011-11-21

**Authors:** Juan M. Murillo-Maldonado, Fouad Bou Zeineddine, Rachel Stock, Justin Thackeray, Juan R. Riesgo-Escovar

**Affiliations:** 1 Departamento de Neurobiología del Desarrollo y Neurofisiología, Instituto de Neurobiología, Universidad Nacional Autónoma de México Campus Juriquilla, Querétaro, Querétaro, México; 2 Biology Department, Clark University, Worcester, Maine, United States of America; Alexander Flemming Biomedical Sciences Research Center, Greece

## Abstract

Coordination between growth and patterning/differentiation is critical if appropriate final organ structure and size is to be achieved. Understanding how these two processes are regulated is therefore a fundamental and as yet incompletely answered question. Here we show through genetic analysis that the phospholipase C-γ (PLC-γ) encoded by *small wing* (*sl*) acts as such a link between growth and patterning/differentiation by modulating some MAPK outputs once activated by the insulin pathway; particularly, *sl* promotes growth and suppresses ectopic differentiation in the developing eye and wing, allowing cells to attain a normal size and differentiate properly. *sl* mutants have previously been shown to have a combination of both growth and patterning/differentiation phenotypes: small wings, ectopic wing veins, and extra R7 photoreceptor cells. We show here that PLC-γ activated by the insulin pathway participates broadly and positively during cell growth modulating EGF pathway activity, whereas in cell differentiation PLC-γ activated by the insulin receptor negatively regulates the EGF pathway. These roles require different SH2 domains of PLC-γ, and act via classic PLC-γ signaling and EGF ligand processing. By means of PLC-γ, the insulin receptor therefore modulates differentiation as well as growth. Overall, our results provide evidence that PLC-γ acts during development at a time when growth ends and differentiation begins, and is important for proper coordination of these two processes.

## Introduction

During development, proper coordination of growth, proliferation, and cellular differentiation is critical for appropriate final organ structure and size. These processes involve signal transduction cascades that determine the spatial and temporal pattern of cellular instructions. Many of these signaling pathways are activated by growth factors via receptor tyrosine kinases (RTKs). In vertebrates, phospholipase C-Γ (PLC-Γ) is required by some RTKs to regulate these cellular processes. PLC-Γ is an intracellular enzyme activated by a multistep process; the initial step involves membrane association via RTKs (or non-receptor tyrosine kinases) through an SH2 (Src-homology 2) domain in PLC-γ with a phosphotyrosine residue in the intracellular domain of the activated receptor. Following this binding, PLC-γ is phosphorylated by the RTK on one or more tyrosines and then catalyzes the hydrolysis of the membrane phospholipid phosphatidylinositol (4,5) bisphosphate (PIP_2_), generating two intracellular messengers: inositol 1,4,5-trisphosphate (IP_3_) and diacylglycerol (DAG). IP_3_ promotes the release of Ca^2+^ from intracellular stores, via its association with the inositol triphosphate receptor (IP_3_R), while DAG, together with Ca^2+^, activates protein kinase C (PKC) [1], [2], [3], [4].

In *Drosophila melanogaster*, a single PLC-Γ gene has been identified, encoded by the locus *small wing* (*sl*) [5]. The mRNA is expressed throughout development, with overall expression highest during embryogenesis [6]. Surprisingly, given this early expression, a large fraction of homozygotes reach adulthood; homozygous *sl* null mutants are recovered, and are viable and fertile. Yet, *sl* homozygotes do have developmental defects: their eyes have a mildly rough phenotype, due to some ommatidia containing one or more extra R7 photoreceptor cells, and their wings are smaller than wild type and contain patches of ectopic veins [5]. These phenotypes indicate that Sl is involved in regulating differentiation of R7 photoreceptor cells and wing veins, as well as growth control in the wing.

Genetic experiments suggest that the extra R7 photoreceptor phenotype results from over activation of the MAPK cascade, showing that Sl is a negative regulator of this pathway during photoreceptor development [5]. *sl* was also identified in a cell-based RNAi screen for loss-of-function phenotypes that inhibit endoplasmic reticulum (ER) accumulation of processed Spitz (Spi), which is an EGF receptor ligand [7]. This suggests that the *sl*-encoded PLC-γ participates in a Spi retention mechanism in the ER. Genetic rescue experiments indicate that Sl is required specifically in cells processing the ligand (a non-cell autonomous effect), as demonstrated by the rescuing capacity in *sl* mutant flies of wild type Sl expression in R8 photoreceptor cells only [7]. Taken together, these results suggest that Sl inhibits the MAPK pathway through controlled retention of Spi in the ER of R8 cells in developing compound eyes.

Nevertheless, other questions remain. What is the mechanism by which Sl regulates these different processes (growth, differentiation), and how is Sl itself activated? Do other RTKs activate Sl, as is the case for PLC-γ in vertebrates? Besides the EGF receptor, an interesting additional RTK candidate for Sl activation is the insulin receptor, because the insulin pathway regulates growth and cellular proliferation in all tissues in *Drosophila* [8]. Most notably, this pathway was recently shown to be involved in controlling the timing of differentiation by modulating EGF/MAPK target genes in developing photoreceptors [9].

Here, we show that Sl stimulates growth. We use the wing to show through genetic experiments that together with the insulin and EGF/MAPK pathways and in a manner dependent on components downstream of Sl such as the IP_3_R and Pkc53E, Sl stimulates cellular growth. Also, in the developing wing and eye, Sl activated by the insulin receptor inhibits MAPK signaling and thus cellular differentiation in a manner again dependent on PLC-γ signaling downstream components, such as the IP_3_R. We also show that PLC-γ genetically interacts with Star and Rhomboid, suggesting modulation of the processing of the EGF ligand Spitz as an avenue of Sl function in all these different phenotypes.

Finally, we also show that the C-SH2 and N-SH2 domains individually are not required for cellular growth, whereas only the N-SH2 domain is required for patterning/differentiation.

## Results

### Sl modulates cellular growth in the wing

**Figure 1:**
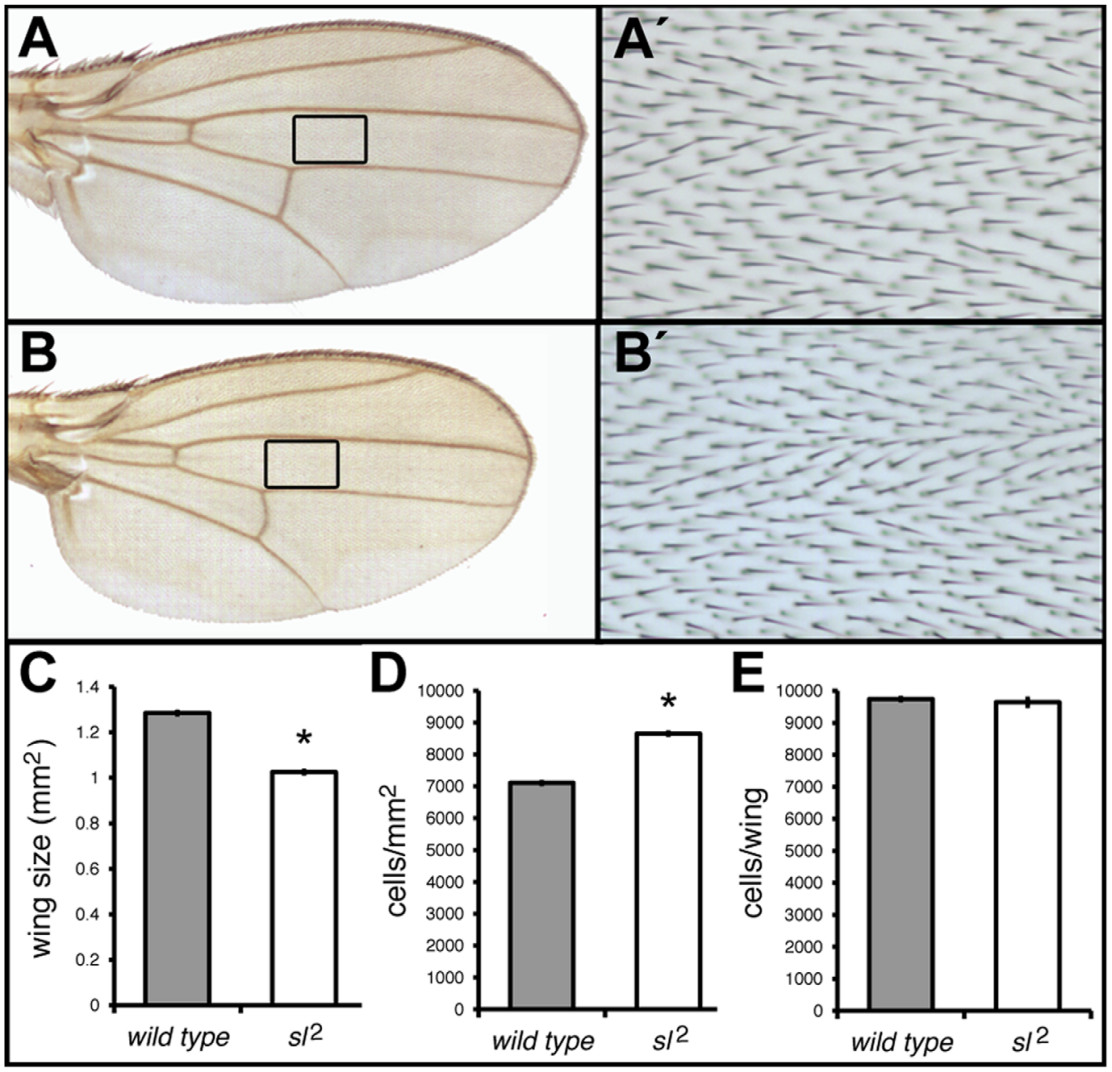
Wing phenotype of *sl* ^2^ mutants compared to ORR (Oregon R) controls. Only male flies were used in this and subsequent figures unless otherwise noted. (A) Wild type wing. (A') shows a higher magnification of boxed area in (A), illustrating the wing region used to determine cell density. (B) *sl* ^2^ mutant wing. (B') shows an amplification of boxed area in (B), used to determine cell density. (C) Histogram showing the reduced mutant *sl* ^2^ wings area. n = 100. (D) Histogram showing an increase in cell density in *sl* mutant wings. n = 100. (E) Histogram showing the averages of total number of cells of complete wing surfaces. n = 5. *sl* ^2^ and ORR do not differ significantly. *p<0.001; error bars represent SEM.

Wings from *sl* ^1^ and *sl* ^2^ mutants were previously shown to be reduced in length by about 20% [5]; although these are not null alleles, they are strong hypomorphs. We compared wild-type and *sl* mutant wings and found that the area of the wing blade is also reduced by 20% (Fig. 1A, B, C). To determine whether this phenotype results from a reduction in growth or cellular proliferation, we determined cell density in an area between veins 3 and 4 in the wing (Fig. 1A and 1B). Mutant wings showed significantly higher cell density than wild-type wings (mean of 8653 and 7106 cells/mm^2^, respectively; Fig. 1A', 1B' and 1D), indicating that the mutant wing blade cells are 21% smaller than wild-type. This means that the reduced area of the wing can be fully explained by a reduction in cell size. Consistent with this model, we found that the number of cells in wild-type and *sl* mutant wings is not significantly different (Fig. 1E). This demonstrates that Sl normally acts to stimulate cell growth in the wing, but does not affect cell proliferation in this tissue.

**Figure 2:**
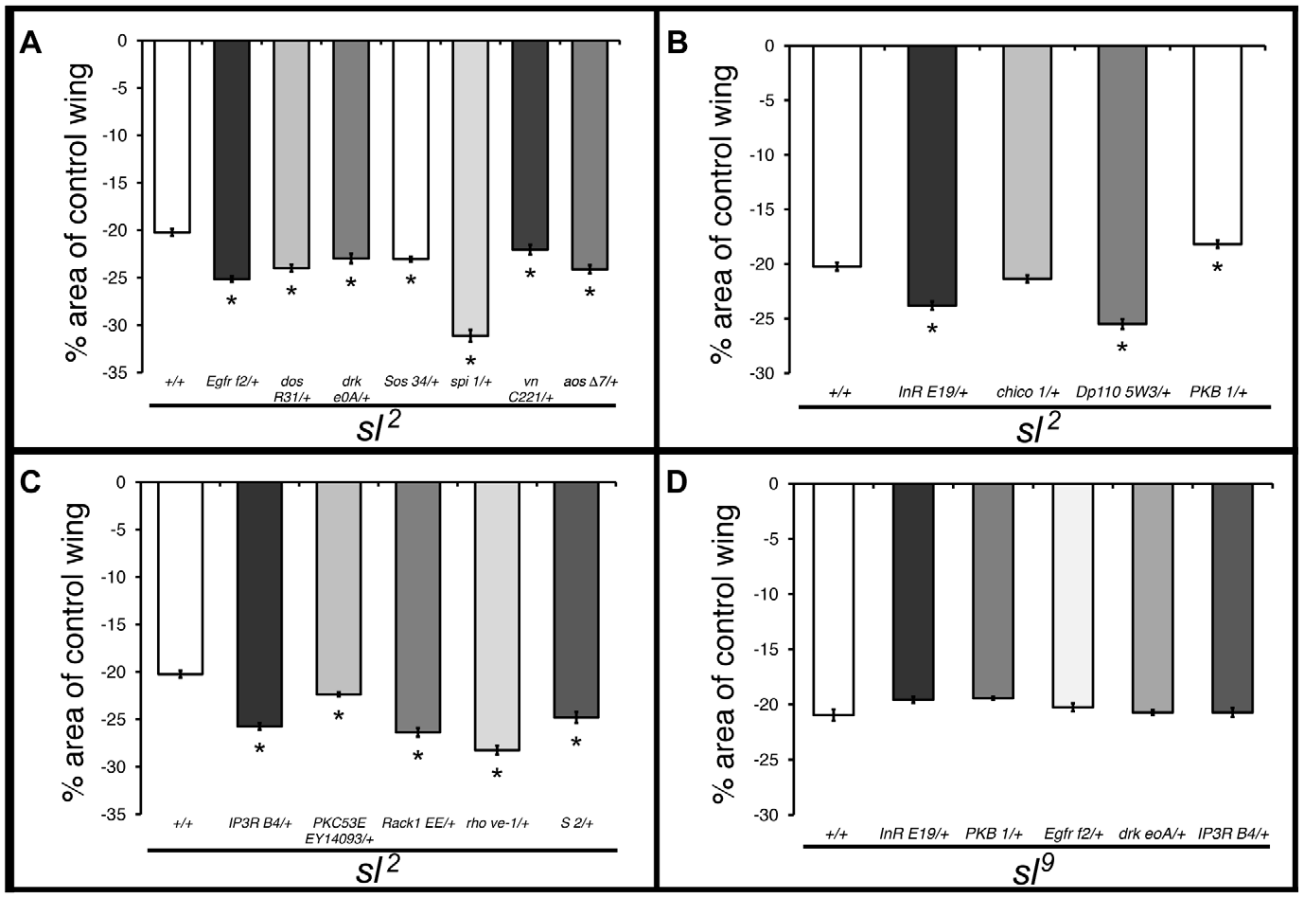
Reduced gene dosage of MAPK and insulin pathway genes on *sl* mutant wings. Heterozygous mutant conditions for EGF/MAPK (A) and insulin (B) signaling genes, and genes downstream of Sl (C) change the *sl* ^2^ mutant wing size. Histogram in (D) shows the effects on *sl* ^9^ mutant wing size of heterozygosity for genes of the insulin and MAPK pathways and of the *IP_3_R*. n = 100 in all cases, * *p*<0.001; error bars represent SEM. In this and subsequent figures showing genetic interactions, tests were done with hemizygous *sl* ^2^ or *sl* ^9^ males and heterozygous or wild type for the genes tested unless otherwise noted.

To determine how Sl stimulates cell growth, we examined the effects of reductions in gene dosage in the EGF/MAPK and insulin signaling pathways on the size of *sl* mutant wings, since both the MAPK [10] and insulin pathways [8] have been shown to regulate growth in the wing. The EGFR has multiple ligands: Spitz (Spi) and Vein (Vn) are activating ligands, whereas Argos (Aos) is inhibitory. Once activated, the EGFR binds to a cytoplasmic scaffold protein, Daughter of sevenless (Dos), and to an adaptor protein, Downstream of receptor kinase (Drk), which leads to the membrane translocation of the guanine nucleotide exchange factor Son of sevenless (Sos). There, Sos favors exchange of bound GDP for GTP in Ras85D, and that, in turn, leads to activation of the MAPKKK Pole hole (Phl; also known as Raf1), which in turn activates the Downstream of raf1 (Dsor1) MAPKK; activated Dsor1 then activates Rolled (Rl), the *Drosophila* MAPK [10]. All mutant *sl* ^2^ flies heterozygous for mutations in MAPK pathway components tested have a small but significant further reduction in the area of the wing blade compared to *sl* ^2^ mutants (Fig. 2A).

In the insulin pathway, the activated insulin receptor (Inr) binds to the cytoplasmic substrate Chico (a Drosophila homolog of IRS), which leads to the activation of the lipid kinase phosphoinositide 3-kinase (PI3K) and, further downstream, the ser/thr kinase protein kinase B (PKB, also referred to as Akt1) [11]. Mutant *sl* ^2^ flies heterozygous for mutations in *Inr* and *Dp110* (the catalytic subunit of PI3K) showed a similar effect to mutations in genes of the MAPK pathway (Fig. 2B). In contrast, *sl* ^2^ flies with a mutation in *PKB* have bigger wings than *sl* ^2^ alone, suggesting a negative feedback loop on pathways stimulating cellular growth in this context. Taken together, these data indicate that Sl normally participates with the EGF/MAPK and insulin pathways as a positive mediator of cell growth in the wing.

PLC-γ catalytic activity generates IP_3_ and DAG by PIP_2_ hydrolysis, leading to modulation of Ca^2+^ release by IP_3_ receptors and PKC activation. In order to study PKC involvement, we characterized a mutation in one of the fly homologs of PKC, *PKC53E. PKC53E* mutants are homozygous viable and fertile, yet have a reduction in wing size of 11.5%, similar to *sl* mutants; by contrast, *PKC53E* heterozygotes have wild-type wings (Fig. S1A). We therefore tested whether the *PKC53E* growth phenotype can be tied to known PLC second messengers. In an *sl* mutant background, reduced gene dosage of *IP_3_R* or *PKC53E* produced further reductions in wing size (Fig. 2C). In the same *sl* mutant background, heterozygosity for a mutation in *Rack1*, a gene that encodes an scaffold protein for various signaling molecules including PKC [12], generated a similar effect (Fig. 2C). These results are consistent with the idea that the cell growth defect in *sl* mutants operates via the classic PLC second messengers IP_3_ and DAG, with a concomitant effect on PKC.

Further downstream, we sought to investigate whether processing of Spitz is required for *sl* dependent wing growth, as Sl has been shown to participate in a Spi retention mechanism in the ER during eye development [7]. To do so, we evaluated the function of *rhomboid* (*rho*), which encodes a Golgi membrane protease that cleaves the inactive, membrane bound Spi precursor to generate mature, secretable Spi [13]; *Star* (*S*), which encodes a type II transmembrane protein involved in trafficking Spi from the ER to the Golgi [13], [14], and *spi* itself. Flies heterozygous for mutations in these three genes in an *sl* ^2^ mutant background indeed showed greater reductions in wing size than solely *sl* ^2^ mutant flies (Fig. 2). Heterozygosity for *spi* in an *sl* mutant background gave the biggest reduction of all tested interactions: wings were reduced a further 65% assuming *sl^2^* reduction as 100%, while heterozygosity for *IP_3_R*, *PKC53E*, *Rack1*, *rho* and *S* gave reductions between 10 and 45%. This suggests that Sl regulates cellular growth in the wing by positively modulating Spi processing. Heterozygosity for *aos* and *vn* also gave further reductions in wing size, indicating their involvement in modulating MAPK pathway activity by *sl* (Fig. 2A).

We repeated some of these genetic interactions with the molecular null allele *sl* ^9^. We found that mutations in the MAPK and insulin pathways, as well as in the *IP_3_R*, do not modify the wing size of *sl* ^9^ mutant flies (Fig. 2D). As *sl^9^* is a molecular null, this means that at the time of Sl function, the MAPK/insulin pathways regulate wing size solely through Sl-dependent avenues.

### Sl has a broad role in cell growth

**Figure 3:**
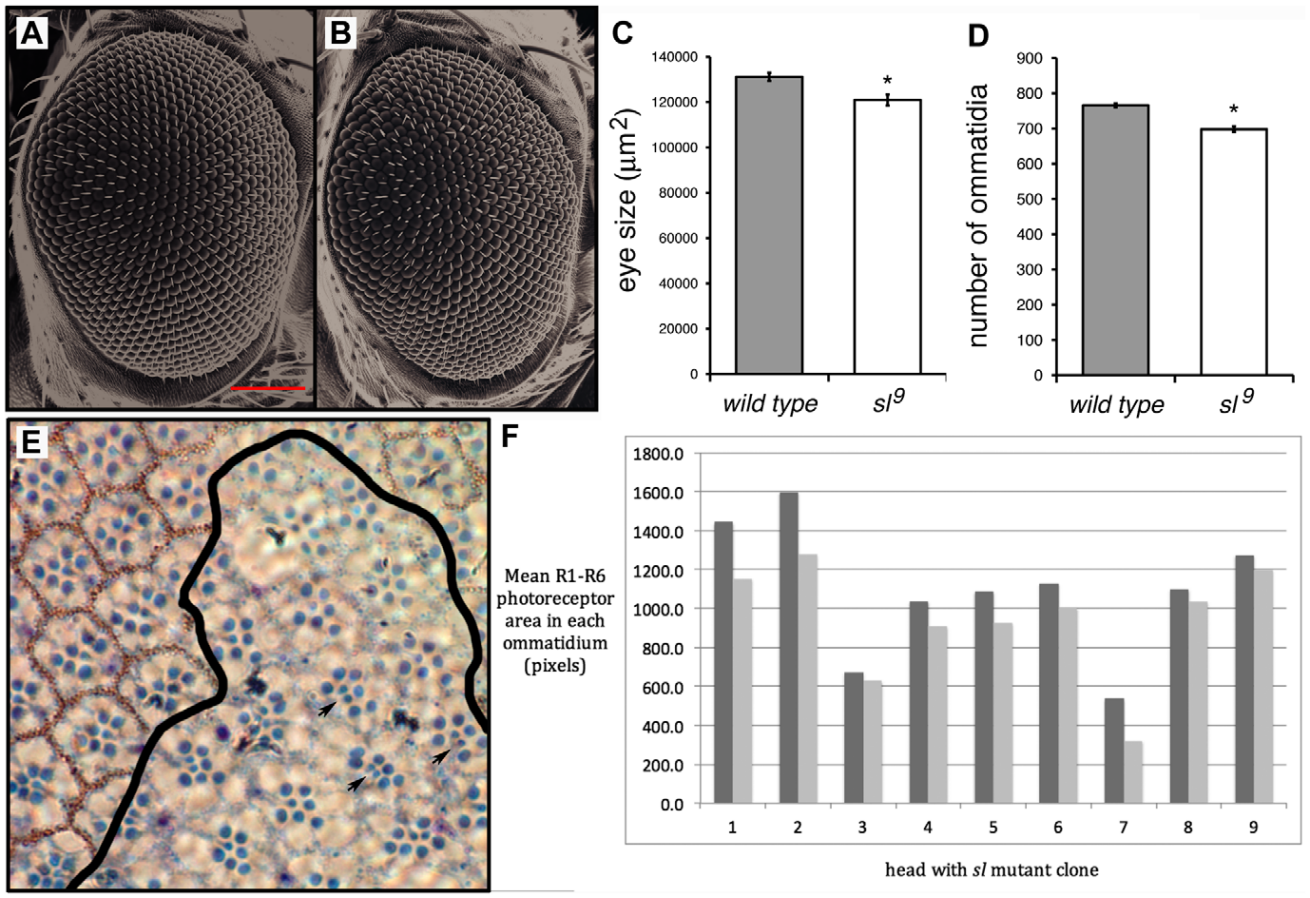
Sl affects eye size. Images in (A, B) are SEMs of control (A; Canton S) and (B) *sl^9^* homozygote female flies. Notice mild roughness of *sl^9^* mutant eye; red bar in A is 100 µm. (C) Average areas of whole eyes of control (Canton S) and *sl^9^* homozygotes. Areas are expressed in µm^2^; n = 18 for Canton S and 16 for *sl^9^*. (D) Average number of ommatidia in eyes of Canton S and *sl^9^* homozygotes (n = 13 for both Canton S and *sl^9^*). Digitized images of eyes were used for measurements in (C) and (D), and in both cases differences are significant at p<0.002 (C) and p<0.0001 (D). (E) Plastic section through an eye containing a *y w sl^9^* homozygous clone, marked by the absence of red pigment surrounding the ommatidia; a black line indicates the approximate edge of the mutant clone. Three ommatidia showing the extra R7 photoreceptors characteristic of *sl* mutants are indicated by arrows. (F) A comparison of *sl* ^+^ and *sl* mutant tissue in nine heads (each pair of bars represents data from an individual head; dark grey bars represent wild-type cells, light grey bars represent cells in mutant patches). The area of R1–R6 rhabdomeres was determined in three to fifteen pairs of nearby ommatidia in each head, each pair consisting of one *sl* ^+^ (*w* ^+^) ommatidium and one *sl* ^9^ homozygous (*w* ^-^) ommatidium.

Since Sl normally acts to stimulate cellular growth in the wing, we wondered whether it plays a similar role in other tissues. As *sl* mutant eyes have extra R7 photoreceptor cells, indicative of *sl* function there [5], we sought to investigate whether growth phenotypes are present in this structure as well. We found that indeed, the area of the eye in *sl^9^* homozygotes is reduced by about 8% compared to wild-type controls (p<0.002; Figs. 3A, B, C). This reduction can largely be explained by a 9% reduction in the number of ommatidia, as we found a mean of 765 ommatidia in wild-type controls compared to only 698 in *sl^9^* homozygous eyes (p<0.0001; Fig. 3D).

Given that *sl* mutants show smaller cells in the wing, we also tested whether there might also be a cell size change in the eye, using the FLP-FRT system to generate mutant clones in otherwise wild-type tissue. Because loss of *sl* results in the recruitment of additional R7 photoreceptors [5], we compared the combined area of the rhabdomeres in R1–R6 photoreceptor cells between adjacent *sl* ^9^/+ and *sl* ^9^ mutant patches. We found that this area was reduced in mutant patches by an average of 15.2% compared to adjacent *sl* ^9^/+ patches in the same section (Figs. 3E and 3F).

If Sl is involved more globally in growth control we might expect to see a change in the overall size of the fly, as observed for mutations in the insulin signaling pathway [8]. One way in which this might be manifest is in the animal's mass. We compared two-day-old *sl^9^* homozygous females with sibling *sl^9^* heterozygotes and found the mutants are about 8% lighter: 1.29±0.019 mg compared to 1.41±0.017 mg. This difference is highly significant (p<0.001; n = 70 per genotype; mean ± SEM). We also evaluated pupae using the same genotypes, measuring both pupal length [15], [16] and weight of stage 12–15 pupae. There were no significant differences (3.16±0.01 mm for *sl^9^* homozygotes to 3.14±0.009 mm for *sl^9^* heterozygotes measuring pupal longest axis, and 1.446±0.012 mg for *sl^9^* homozygotes, to 1.464±0.012 mg for *sl^9^* heterozygotes. n = 73 for *sl^9^* homozygotes, and n = 79 for *sl^9^* heterozygotes; mean ± SEM). These analyses show that Sl plays a more general role in the promotion of cell growth, beyond the one classically described in the wing.

### Insulin receptor via Sl inhibits wing vein differentiation

**Figure 4:**
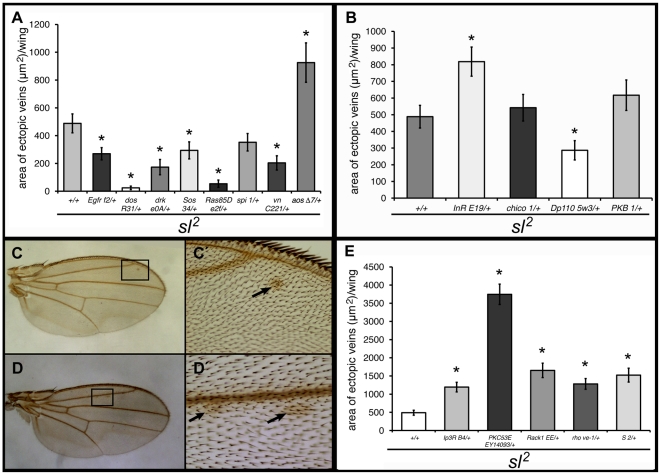
Reduced gene dosage of MAPK and insulin pathway genes on *sl^2^*ectopic wing veins. Shown are effects of heterozygous mutant conditions for EGF/MAPK (A) and insulin (B) pathway genes on the *sl* ^2^ ectopic vein phenotype. (C, D) show wings of heteroallelic mutant *InR* ^E19/3T5^ (C) and *PKB* ^1/3^ (D) flies with small ectopic vein-like patches. (C') and (D') show close-ups of boxed areas in (C) and (D), respectively. Arrows point to vein-like material present. As heterozygotes, neither *InR* nor *PKB* show ectopic wing vein-like material. (E) Effects of heterozygosity for *IP_3_R* ^B4^, *PKC53E* ^EY14093^, *Rack1* ^EE^, *rho* ^ve-1^ and *S* ^2^ on the extent of *sl^2^* ectopic wing veins. n = 100. *p<0.001; error bars represent SEM.

*sl* ^2^ mutant flies sometimes show small ectopic wing veins, indicating an excess of cellular differentiation [5]. Vein differentiation in the wing is regulated by various pathways, including the EGF/MAPK, amongst others [review in 17,18]. We found that reductions in gene dosage of most MAPK pathway genes tested partially rescued the ectopic vein phenotype of *sl* ^2^ mutant wings, indicating that the ectopic veins are a result of over activation of MAPK signaling, the exceptions being *aos* and *spi*. Consistent with its role as an EGFR inhibitory ligand, heterozygosity for *aos* significantly augmented the ectopic vein *sl* phenotype, strongly implying Aos in the regulation of vein differentiation. In contrast, heterozygosity for *spi* had no significant effect, indicating that for vein differentiation Vn, rather than Spi, is critical (Fig. 4A).

*sl* ^2^ mutant flies heterozygous for mutations in the insulin pathway show no consistent change in the ectopic veins phenotype of *sl* ^2^ wings; one shows a significant increase (*InR*), one shows a significant decrease (*Dp110*) and two others (*chico* and *PKB*) are unchanged (Fig. 4B). Given that the *sl* ectopic vein phenotype is exacerbated in a heterozygous *InR* background, this argues that the *sl*-encoded PLC-γ requires the insulin receptor to regulate cell differentiation in the wing, but the pathway downstream of the receptor may not be the standard one described in other contexts.

To explore further the possibility of a role for insulin signaling in wing vein formation we examined viable heteroallelic combinations of both *InR* and *PKB* (*InR* ^E19/3T5^ and *PKB* ^1/3^). In each case we observed low percentages of wings with small ectopic vein-like patches (12.7%, n = 79 for *InR* and 8% n = 87 for *PKB*), confirming their participation in vein differentiation (Fig. 4C and 4D).

We wondered whether the excess vein differentiation in the *sl* mutants occurs via the canonical downstream PLC effectors, so we determined the effect of changes in dosage of some of these. Heterozygosity for mutations in the *IP_3_R* and *Rack1* significantly increased the ectopic vein area in *sl* ^2^ mutant wings; in addition, we observed a dramatic increase in the vein phenotype when the dosage of *PKC53E* was reduced in an *sl^2^* mutant background (Fig. 4E). This is consistent with a previous report describing ectopic expression of the PKC53E kinase domain, suggesting that PKC53E regulates vein differentiation in the wing [19]. In agreement with this finding, we found that flies homozygous for a hypomorphic allele of *PKC53E* also show ectopic veins (Fig. S1B), even if surveyed in an *sl* ^+^ background. This argues that function of this PKC homolog, PKC53E, is particularly important for vein formation.

As mentioned earlier, the mechanism by which Sl affects R7 photoreceptor differentiation is proposed to be via its involvement in ER retention of the EGF ligand Spitz, in a pathway that also involves Rho and Star [7]. Does the same mechanism operate in the wing? We found that heterozygosity for mutant alleles of *rho* and *S*, but not *spi*, increased the vein phenotype of *sl* ^2^ mutants (Fig. 4E), suggesting a Spi-independent role for Rhomboid-1 and Star in modulation of vein differentiation, perhaps by their involvement in the processing of a MAPK pathway ligand other than Spi, such as Keren.

We looked at some of the same interactions with the null allele *sl* ^9^ and found that mutations in the MAPK and insulin pathways in general, as well as in the *IP_3_R*, affect the extent of ectopic vein territory in *sl* ^9^ mutant flies in a similar fashion to that seen in *sl* ^2^ mutants (Fig. S2). Because a further increase in severity of the wing vein phenotype can be seen in *sl^9^*, this indicates that Sl is not the only avenue of vein differentiation modulation by insulin signaling. IP_3_R activity is also required independent of Sl, most likely regulated by cytoplasmic calcium concentrations as the IP_3_R is known to be regulated by calcium [20], and this could account for the exacerbation of the ectopic vein phenotype in an *sl^9^* null mutant background. Taken together, these results confirm a previously unrecognized role for insulin receptor signaling in differentiation and imply that there are both Sl-dependent and Sl-independent ways of regulating vein differentiation by insulin signaling in the wing.

### Insulin receptor participates in R7 photoreceptor differentiation via Sl

**Figure 5:**
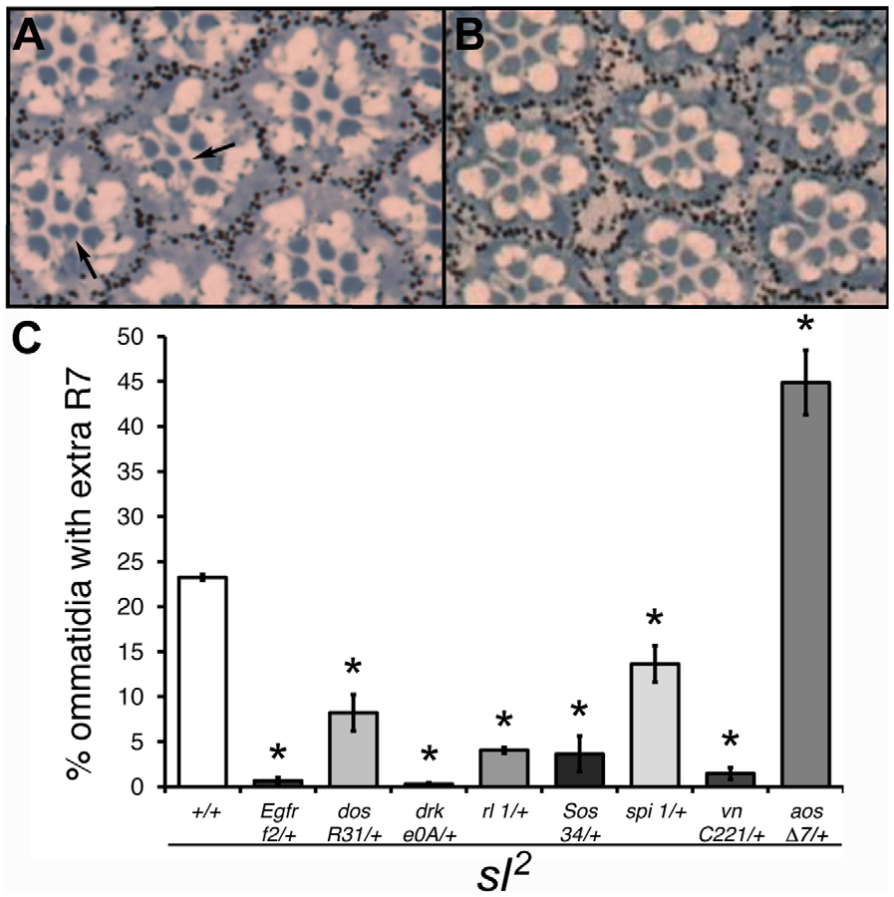
Reduced gene dosage of MAPK pathway genes on *sl* ^2^ R7 phenotype. Tangential sections of the distal part of eyes from *sl* ^2^ (A) and *sl* ^2^ heterozygous for *Drk* ^e0A^ (B) flies, stained with toluidine blue. The arrows indicate extra R7 cells. (C) Histogram showing the effect of heterozygosity for mutations in genes of the MAPK pathway on extra R7 cells in *sl* ^2^ mutants. n = 5 eyes each with ≤150 ommatidia per eye. *p<0.001; error bars represent SEM.

*sl* mutant flies have extra R7 photoreceptors in the eye, indicating that Sl normally inhibits cellular differentiation of this cell type [5]. A reduction in gene dosage of components of the MAPK pathway partially rescues the extra R7 photoreceptor phenotype in *sl* mutant flies ([5] and Figs. 5B and 5C), indicating that the differentiation defect, like the ectopic vein phenotype, is due to pathway over activation. The sole exception, of the genes tested, is *aos*. As expected, and consistent with its role as an EGFR inhibitory ligand, heterozygosity for *aos* in an *sl* mutant background resulted in a dramatic increase in the *sl* mutant extra R7 phenotype. Male *sl^2^; aos* ^Δ*7*^ */+* eyes also have noticeably increased roughness (Fig. S3).

**Figure 6:**
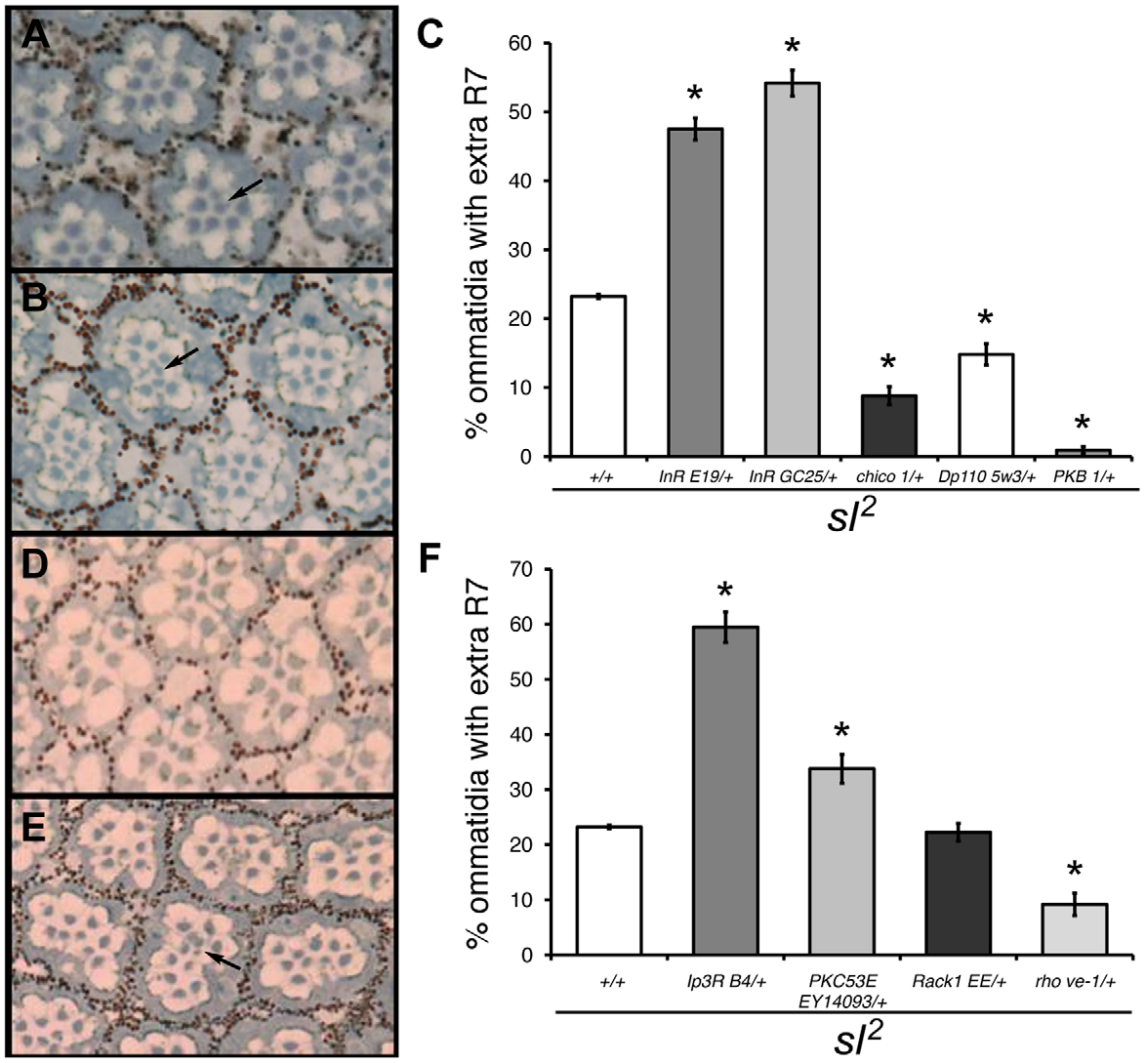
Reduced gene dosage of insulin pathway and downstream components on the *sl^2^* extra R7 phenotype. Tangential sections of the distal part of eyes from *sl* ^2^ (A) and *sl* ^2^ heterozygous for *InR* ^E19^ (B) flies, stained with toluidine blue. Arrows indicate extra R7 cells. (C) Histogram showing the effect of heterozygosity for mutations in genes of the insulin pathway on the percentage of ommatidia with extra R7 cells in *sl* ^2^ mutants. n = 5 eyes each with ≤150 ommatidia per eye. *p<0.001; error bars represent SEM. Tangential sections of the distal part of eyes from *sl* ^2^ (D) and *sl* ^2^ heterozygous for *Ip_3_R* ^B4^(E) flies, stained with toluidine blue. Arrow indicates ectopic R7 cells. (F) Histogram showing the effect of heterozygosity for *Ip_3_R* ^B4^, *PKC53E^EY14093^*, *Rack1^EE^* and *rho* ^ve-1^ on the percentage of ommatidia with extra R7 cells in *sl* ^2^ mutants. n = 5 eyes each with ≤150 ommatidia per eye. *p<0.001; error bars represent SEM.

We examined next whether the insulin pathway is also involved in this process by analyzing the effect of heterozygous mutations in this pathway on the number of ommatidia with extra R7 cells in *sl* ^2^ mutant eyes. Similar to our findings with the ectopic wing vein phenotype, only heterozygosity for mutations in the insulin receptor itself showed a significant increase in the photoreceptor phenotype (Fig. 6B and 6C). The average number of R7 cells per ommatidium rose dramatically: from 1.25±0.003 in *sl^2^* males to 1.89±0.36 (n = 4–6 eyes, p<0.002) for sibling *sl^2^* males with a copy of *InR^GC25^*. We found the same result with another hypomorphic allele of PLC-γ: *sl^1^* (Fig. S4A). The average number of R7 cells per ommatidium rose from 1.10±0.037 in *sl^1^* males to 1.58±0.038 (n = 5–7 eyes, p<0.002) for sibling *sl^1^* males with a copy of *InR^GC25^*. By contrast, in an *sl^9^* background, the *InR* mutation did not increase the severity of the R7 phenotype (Fig. S4B), but given that *sl^9^* is a molecular null, this result is expected if Sl is the only avenue of modulation for the insulin receptor in the eye. This contrasts with the vein differentiation results, suggesting different modes of activity for Sl in these processes. We also found that reduced dosage of *chico*, *Dp110* and *PKB* significantly decreased the extra R7 phenotype of *sl* ^2^ mutants (Fig. 6C).

Heterozygosity for a mutation in the *IP_3_R* in an *sl^2^* mutant background showed an increase in the number of ommatidia with extra R7 cells (Fig. 6F), and in the number of R7 cells per ommatidium (Fig. 6E) (from 1.25±0.003 in *sl^2^* males, to 1.76±0.11 in sibling *sl^2^* males with a copy of *IP_3_R^B4^*, n = 5, p<0.001). A similar effect was observed for the same *IP_3_R* mutation in an *sl* ^9^ mutant background (Fig. S4B), indicating that the IP_3_R regulates R7 differentiation in Sl-dependent and Sl-independent ways, as is the case for ectopic vein differentiation. Mutant *sl* ^2^ flies heterozygous for a mutation in *PKC53E* also showed a significant increase in the extra R7 photoreceptor phenotype (Fig. 6F). However, flies homozygous for a *PKC53E* mutation alone showed <1% of ommatidia with extra R7 cells (Fig. S1C); in contrast, about 50% of these mutant flies showed ectopic veins in the wing, indicating that this protein has an important role in the regulation of vein differentiation but not in R7 differentiation. In agreement with this, heterozygosity for *Rack1* did not modify the number of extra R7 cells in an *sl^2^* mutant background (Fig. 6F).

We found that heterozygosity for *rho* and *spi* mutant alleles produced a significant decrease in the extra R7 phenotype of *sl* ^2^ mutants, from 1.25±0.003 in *sl^2^* to 1.09±0.019 for *sl^2^* heterozygous for *rho* and to 1.13±0.015 for sl*^2^* heterozygous for *spi* (Fig. 6F and 5C show these reductions as percentages). *S* mutations have a dominant phenotype in the eye; one of these is a discrete *loss* of R7 cells in *S^2^* (4.97±0.42 percent of ommatidia without R7 cells or 0.95±0.004 R7 cells per ommatidium). We examined whether this dominant phenotype could be modified in an *sl* mutant background, and found that in *S* ^2^ heterozygotes the R7 phenotype is not changed if also mutant for *sl* ^2^ (Fig. S5).

Overall, these data are consistent with a role for Sl in a Spi retention mechanism in the ER in photoreceptor cells [7], suggesting that Sl normally inhibits R7 differentiation by negatively modulating processing of Spi, which in turn modifies the strength of MAPK signaling. In summary, these results suggest that Sl, activated by the insulin receptor, modulates photoreceptor differentiation via the IP_3_R and PKC53E through negative regulation of the EGF/MAPK pathway ligands.

### Role of SH2 domains in Sl function

**Figure 7:**
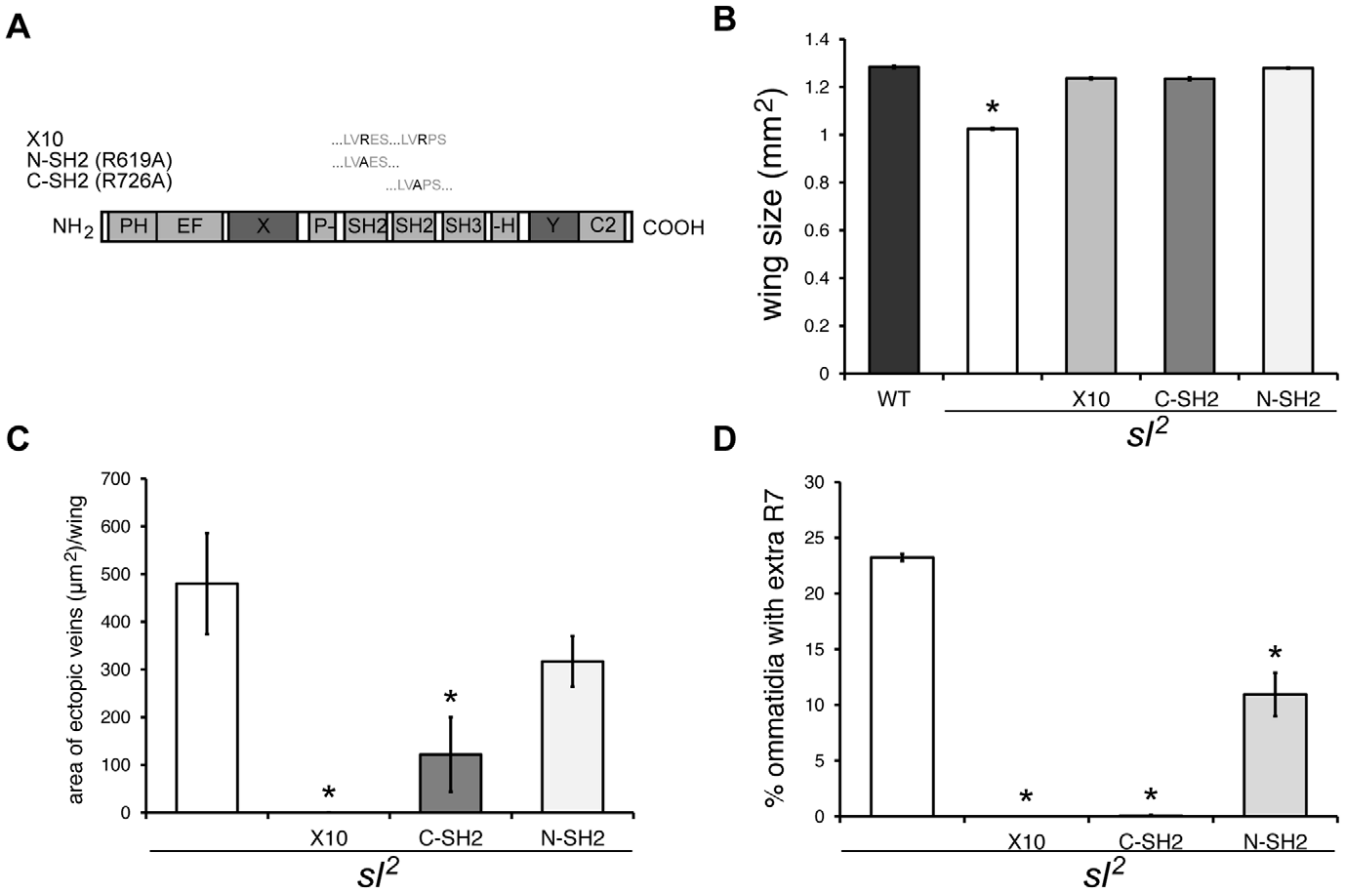
Expression of wild type (X10) and mutant *sl* constructs in a mutant *sl* ^2^ background. (A) Schematic representation of mutations in the SH2 domains of PLC-γ. (B) Histogram showing the effect of expression of *sl* constructs on *sl* ^2^ wing size. n = 100. *p<0.001; error bars represent SEM. (C) Histogram showing the effect of expression of *sl* constructs on ectopic wing veins of *sl* ^2^ mutant flies. n = 100. *p<0.001; error bars represent SEM. (D) Histogram showing the effect of expression of *sl* constructs on extra R7 cells in *sl* ^2^ mutant eyes. n = 5 eyes each with ≤150 ommatidia per eye. *p<0.001; error bars represent SEM.

In order to study in more detail the relationship between Sl and receptor tyrosine kinases (insulin and EGF receptors) in the wing and eye, we examined the role of the Sl SH2 domains, which are known to be involved in the binding of PLC-γ to RTKs in vertebrates. We generated *sl* genomic rescue constructs with either the N- or C-terminal SH2 domains carrying mutations that disable their function in other systems (Fig. 7A) [21], [22]. Transgenic flies containing a wild type *sl* construct (X10), C-SH2 mutant (C-SH2) or N-SH2 mutant (N-SH2) constructs rescued the wing size defect in *sl* ^2^ mutants (Fig. 7B). However, while the wild type and C-SH2 mutant constructs rescued the ectopic veins and extra R7 photoreceptors phenotype, the N-SH2 mutant construct did not (Figs. 7C and 7D). These results are consistent with experiments in mammals, in which the two SH2 domains have differential roles in the binding and activation of PLC-γ by RTKs[21].

## Discussion

### Sl modulates cellular growth in the wing

**Figure 8:**
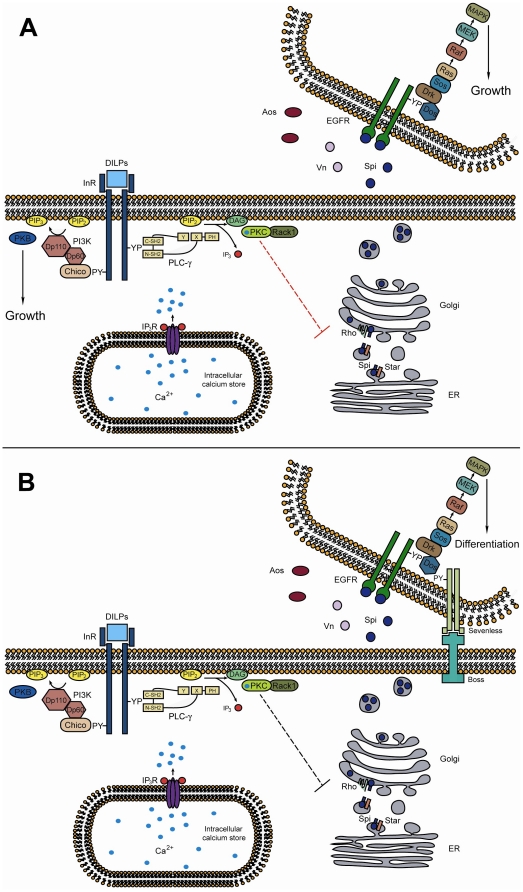
Sl modes of action in growth and differentiation. Panel (A) shows Sl, activated by the insulin pathway, acting as a liaison regulating MAPK pathway ligand processing, to foster MAPK activation to a level promoting growth (red inhibitory interaction). (B) Conversely, for differentiation, reduced insulin receptor signaling leads to lower levels of Sl activation and augmented Spi processing (different from A, gray inhibitory interaction; possibly other targets from those in A), and this, in turn, allows MAPK activation in a manner consistent with promotion of differentiation.

We show, by measuring cell density, that *sl* mutant wings have a reduction in cell growth but not cell proliferation. This defect is qualitatively similar to mutations in MAPK signaling; cells with homozygous mutations for members of this pathway have higher cell densities, suggesting smaller cells [10]. Of the several signaling pathways known to be involved in *Drosophila* wing growth [23], only the MAPK and insulin pathways are triggered by tyrosine kinase receptors that are likely to activate Sl. Our results show that indeed both pathways are genetically linked to Sl in promoting cell growth, probably acting in a concerted fashion; further molecular studies will be required to reveal the molecular mechanisms and physical interactions that allow this link. Sl signaling thus provides a means for coordinating growth by forming a regulatory link between the MAPK and insulin pathways. In this scenario, Sl activated by the insulin pathway would function by modulating MAPK output; that is to say, to reduce somewhat the levels of MAPK activity, but not to stop it, as no MAPK activity leads to no growth and cell death [10], [24], and too much MAPK activity leads to ectopic differentiation and reduced growth (our results, and [7]) (Fig. 8A).

### Sl modulates cellular growth in the eye

We found that Sl regulates cellular growth in the eye. Whole eyes are smaller, and the difference in size can be largely explained by the presence of fewer ommatidia. This means that *sl* mutant eyes very likely contain fewer cells, despite the fact that some ommatidia sport one or two extra R7 cells, as the number of cells missing due to reduced numbers of ommatidia is bigger than the number of extra R7 cells present. This suggests either reduced proliferation or increased cell death in differentiating *sl* mutant eyes, and is different from the growth defect found in wings, yet consistent with a moderate requirement of MAPK output to promote growth and cellular survival.

We also surveyed whether mutant eye cells are smaller, in a situation where a direct comparison could be made between mutant and wild-type cells. By studying *sl* loss-of-function clones in the eye, we determined that *sl* mutant cells are smaller than neighboring wild-type cells. This growth defect is similar to that seen in wings, suggesting a more general growth regulation by Sl in different tissues, albeit in a situation where competition between cells of different genotype does take place.

### Sl also has growth effects beyond the eye and wing

Not only is cell size reduced to a similar extent in both the eye and wing of *sl* homozygotes; we found that the adult animal as a whole has reduced mass. Given that the reduction in mass (8%) is of a similar magnitude to the reduction in cell size in the eye (15%) and wing (20%), the most parsimonious explanation for this change in mass is that the same Sl functions found in the eye and wing are required more generally throughout the animal, suggesting that cell size may be reduced in many tissues. However, we found that the reduced growth observed in the adult was not reflected by a reduction in length of *sl* mutant pupae. This is in contrast to mutations of other genes involved in growth control, such as the neurofibromin 1 gene [15], [16], which shows a significant reduction in pupal length. This might be because *sl* has a relatively small effect on growth, varying between 5% and 20% in different contexts, so our sample may not have been large enough to observe a small change in mean length. Given that Sl does not appear to affect the length of appendages other than the wing (J.Thackeray, unpublished data), it may be that there are other compensatory effects resulting from lost Sl function that maintain the pupal case at an approximately wild-type length.

Another complementary explanation for the reduction in adult mass is via a role for Sl on nutrient sensing. As Sl is clearly involved in insulin signaling, and as insulin is required for integrating nutrient sensation in *Drosophila* [25], the effect on mass might be a combination of impacts on both growth signaling and nutrient sensing.

We propose that the overall role for Sl is to act as a pro-growth agent, allowing cells and tissues to attain normal numbers and sizes. This is achieved by dampening MAPK output in growth control in a non-cell autonomous manner, by restricting processing of EGFR ligand(s), as shown previously for R7 cell differentiation [7]. Since both the MAPK and insulin pathways initially act to favor proliferation and growth, we propose that Sl functions here under insulin pathway control, allowing growth to continue, preventing ectopic differentiation (Fig. 8A). There are several ways in which it could do so: by directing activated MAPK to a different cellular compartment (cytosolic versus nuclear [26]) or by controlling overall strength and duration of signaling [27], examples of which have been shown to elicit such changes in developing wing cells in both *Drosophila* and PC12 cells.

### Sl inhibits wing vein differentiation

As opposed to promoting wing cell growth, Sl inhibits wing vein formation. Positioning and differentiation of provein and intervein territories in the wing are determined by at least five different signaling pathways: Hedgehog (Hh), Decapentaplegic (Dpp, a bone morphogenetic protein homolog), epidermal growth factor (EGF)/MAPK, Wingless (Wg; Wnt), and Notch [17]. Genes of the EGF/MAPK signaling pathway are expressed at higher levels in provein territories; activation of this pathway is required for provein to vein differentiation [28]. As mentioned above, *sl* mutant flies show ectopic vein patches indicating an excess of vein differentiation. Here we show that heterozygosity for MAPK pathway genes, in a sensitized *sl* mutant background, caused a partial rescue of the *sl* vein differentiation defect, indicating that the *sl* phenotype is a consequence of MAPK over activation. In this case, Sl acts as a negative regulator of MAPK signaling.

The dual roles played by Sl in the wing via the same MAPK pathway – promotion of growth and subsequently, promotion of differentiation –– can be explained if Sl is helping to establish appropriate MAPK activity at each developmental stage. Alternatively, given that growth and differentiation tend to be temporally separated [29], these distinct effects probably occur in cells that are at different stages of development and therefore different effector molecules are likely to be present. Furthermore, these results also suggest that PI3K (of which Dp110 is a component), which inhibits Sl ectopic veins, might have a negative role in regulation of PLC-γ (figure 4B), in addition to the positive effect described in mammals [30]. Given that PI3K and Sl compete for the same substrate, the interaction we see between them in the wing might due to an altered ratio of phosphatidyl bisphosphates to phosphatidyl trisphosphates available at the membrane near the activated RTK.

### Insulin receptor involvement in wing vein differentiation

Strikingly, there appears to be no role for the insulin pathway in vein differentiation, save for the insulin receptor itself; however, this finding is consistent with studies showing that the insulin pathway as a whole is generally not involved in differentiation [8]. Despite this, we found that mutations in the insulin receptor generate dramatic increases in ectopic wing veins in *sl* mutant backgrounds. These results clearly indicate a requirement for the insulin receptor in Sl-mediated inhibition of vein differentiation, suggesting that Sl is activated by this receptor. In mammalian cells there is some evidence for this type of association [31], but this is the first evidence of such an interaction in *Drosophila*. Such an interaction might be direct via one of the Sl SH2 domains, or indirectly through an adaptor such as Lnk.

### The insulin receptor inhibits R7 photoreceptor differentiation via Sl

Photoreceptor differentiation begins in the third larval instar with the passage of the morphogenetic furrow across the eye imaginal disc. The R8 photoreceptor differentiates first, nucleating developing ommatidia without requiring EGF/MAPK activation. In contrast, the R7 photoreceptor is the last to differentiate and requires MAPK-mediated activation involving both the EGF and Sevenless receptors [32], [33]. The presence of extra R7 photoreceptors in *sl* mutant eyes indicates that Sl normally inhibits R7 differentiation, resulting in only one R7 cell per ommatidium [5]. We confirmed that this phenotype is a consequence of over activation of MAPK signaling, because heterozygous mutant conditions for genes in this pathway almost completely rescue the *sl* eye defect [5], [7]. We also found a role for insulin signaling in R7 development, but as in vein differentiation, only mutations for the insulin receptor itself augmented the *sl* phenotype in an *sl* mutant background. Thus, in this instance as well, the insulin receptor appears to activate Sl to enable it to regulate R7 differentiation. This eye phenotype therefore follows the same logic as vein differentiation, constituting another case of close association between the insulin receptor and Sl. As Sl does not show the same interaction with Chico as with InR, activation of Sl via InR may require either direct binding of Sl to InR or another insulin pathway adaptor molecule, such as Lnk [34]. It will be of interest to see if there is physical interaction between Sl and InR or Lnk in this context. Taken together, these results are consistent with Sl acting to reduce MAPK output to prevent ectopic differentiation (Fig. 8B).

### Sl mode of action

A central function of all phospholipase C enzymes is hydrolysis of PIP_2_. In this study we showed that regulation of growth and differentiation by Sl must depend on PIP_2_ hydrolysis to some extent, because of the interaction between *sl* and mutations in *IP_3_R*, *PKC53E* and *Rack1*. Also, by means of genetic tests, we found that Sl requires the Spi processing machinery (S, Rho) to regulate growth and differentiation. It has previously been shown that Sl acts on Spi processing during R7 differentiation, by favoring Spi retention in the endoplasmic reticulum [7]. In order to rationalize Sl function in all the phenotypes studied, we reason that by inhibiting Spitz processing, Sl could delay initiation of differentiation, allowing still undifferentiated cells to grow and attain a normal size before the onset of differentiation. Sl modes of action in growth and differentiation may be different; *sl* alleles affecting the wing but not the eye is strong evidence for this assertion [35].

In general, during growth, Sl activated by the insulin pathway acts as a liaison regulating MAPK pathway ligand processing, to promote MAPK activation to a level permitting growth. In agreement with a well-characterized case in mammalian cells [27], we propose that this level of activity of MAPK is different from the level required for differentiation; either it is of a different duration, or of an overall different stimulation level, or happening at a different time. Alternatively it occurs in a different subcellular compartment from that required for differentiation [26], acting thru Sl regulation of Spi processing (Fig. 8A, red inhibitory interaction). This scenario also requires both the MAPK and the insulin pathways to be active for cellular growth. Conversely, for differentiation, reduced insulin receptor signaling leads to altered (lower) levels of Sl activation and augmented Spi processing (Fig. 8B, different from Fig. 8A, gray inhibitory interaction; possibly other targets from those in Fig. 8A), and this in turn allows MAPK activation in a manner consistent with promotion of differentiation. This could either be caused by longer or stronger MAPK stimulation, as documented for PC12 cells [27], since lower Sl activity now allows higher levels of MAPK ligand processing, and/or by compartmentalization of the activated MAPK pathway, as shown for the Drosophila wing [26], besides happening at different times during development. In this second case, only the MAPK pathway is required to be fully active. Finally, loss-of-function mutant conditions for *sl* lead to ectopic differentiation at the expense of growth.

We found that genomic *sl* constructs carrying either a mutant C-SH2 or N-SH2 domain rescued the wing size defect of *sl* mutants, to an extent equivalent to a wild type *sl* construct. The amino acid sequence of Sl is well conserved compared to mammals, suggesting that the mode of enzyme activation has not changed since flies and mammals diverged; if this is so, our results imply that PIP_2_ hydrolysis is not required for Sl's role regulating cell growth. This is consistent with previous reports showing that mitogenic activity triggered by PLC-γ in mammals does not depend on its phospholipase function [36]. In mammals, in some contexts, this is due to an SH3-mediated interaction between PLC-γ and PIKE (PI3K enhancer) for nuclear PI3K activity [37]. Another mammalian study showed that the PLC-γ PH domain is sufficient for both membrane association and activation of mammalian PLC-γ following activation of PI3K [30]. If any of these scenarios also occur in Drosophila, this would be consistent and explain why the N-SH2 domain of Sl is dispensable for its role in regulating cell growth, while providing a possible tie-in with the PI3K activation triggered during insulin signaling.

In contrast to the results for cell growth, we found that the N-SH2 domain is necessary for Sl to regulate proper differentiation of R7 photoreceptors and wing veins. In mammals, the PLC-γ N-SH2 domain binds to a phosphotyrosine either on the activated receptor or an adaptor; whereas the PLC-γ1 C-SH2 domain is thought to be necessary for activation of phospholipase activity, binding to a phosphotyrosine within PLC-γ itself, this site having been phosphorylated before by the activated RTK after PLC-γ binding [38]. This suggests that differentiation depends on the more traditional model of PLC-γ activity, requiring binding of the N-SH2 domain to either a receptor or an adaptor. The SH2 mutant constructs therefore confirm earlier findings that the growth and differentiation defects seen in *sl* mutants are driven by distinct and mutationally separable pathways [35].

### “Parental control” by Sl

Taken together, our results indicate that Sl participates in fine coordination of growth and differentiation during development. Although Sl is not essential for wing or eye growth and development, it is necessary to achieve appropriate final structure and size. In the absence of Sl function, these tissues arrest growth prematurely and probably initiate differentiation earlier, resulting in ectopic differentiation while attaining smaller cellular sizes. As such, Sl can be seen as exerting a kind of “parental control” that protects cells from differentiating before attaining a normal size. This function requires Sl to change cellular behavior from growth (or possibly inhibition of differentiation) to differentiation in a short period of time.

PLC-γ1 has been demonstrated to be a phosphorylation target of MAPK [39], [40], and some PKC isoforms can phosphorylate PLC-γ without affecting PIP_2_ hydrolysis [41] so it is clear that there is a complex interplay of signaling among this set of molecules following RTK activation. Further study of the dynamics of Sl-regulated EGF/MAPK signaling in space and time during wing and eye development in Drosophila may help to expose more of this network.

## Materials and Methods

### Drosophila stocks

Flies were raised at 25°C under standard conditions. Fly stocks used were: *sl* ^1^/FM7c and *sl* ^2^/FM7c obtained from J. Carlson (Yale University, USA). The *sl^1^* allele is produced by a insertion of a *412* transposable element in the exon encoding the C-terminal end of the split PH domain, resulting in loss of the region Y catalytic domain as well as the C2 domain; *sl^2^* carries a 13 bp deletion just before the exon encoding the N-terminal SH2 domain, resulting in the loss of both SH2 domains, the SH3 domain, region Y and the C2 domain. Both alleles are strong hypomorphs [35]. *sl^9^/*FM7c was generated in a Canton S background and characterized by one of us (J. Thackeray); *sl^9^* is a point mutation that changes a tryptophan to a stop codon at the beginning of the open reading frame, and thus, creates a null allele [35], [42]. *InR* ^E19^/TM3, *InR* ^GC25^/TM3, *InR* ^3T5^/TM3, *chico* ^1^/*CyO*, *Dp110* ^5W3^/TM6, *PKB* ^1^/TM3 and *PKB* ^3^/TM3 were obtained from E. Hafen (ETH, Switzerland); *aos* ^Δ*7*^ */*TM3, *Egfr* ^f2^/*CyO*, *Dos* ^R31^/TM3, *drk* ^e0A^/*CyO*, *rl* ^1^/*CyO*, Ras85D^e2f^/*CyO*, Sos^34^/*CyO*, Sos^dm7^/*CyO*, *Rack1^EE^/CyO*, *rho^ve-1^*/TM3, *S* ^2^/In(2LR)bw^V1^ds^33K^, *spi^1^/CyO*, *Ip3R* ^B4^/TM3, *PKC53E* ^EY14093^/*CyO, vn^C221^/TM3* and P{*ey*-FLP.N}6, *ry^506^* were from the Bloomington *Drosophila* stock center at Indiana University, USA.

### Genetics

Genetic interactions were made by crossing *sl* ^1^, *sl* ^2^, or *sl* ^9^ homozygous virgin females with males of the following genotypes: *InR* ^E19^/TM3, *InR* ^GC25^/TM3, *chico* ^1^/*CyO*, *Dp110* ^5W3^/TM6, *PKB* ^1^/TM3, *aos* ^Δ*7*^ */*TM3, *Egfr* ^f2^/*CyO*, *Dos* ^R31^/TM3, *drk* ^e0A^/*CyO*, *rl* ^1^/*CyO,* Ras85D^e2f^/*CyO*, Sos^34^/*CyO*, Sos^dm7^/*CyO*, *Rack1^EE^/CyO*, *rho^ve-1^*/TM3, *S* ^2^/In(2LR)bw^V1^ds^33K^, *spi^1^/CyO*, *Ip3R* ^B4^/TM3, *vn^C221^/TM3* and *PKC53E* ^EY14093^/*CyO*. We selected male progeny hemizygous for *sl* ^1^, *sl* ^2^ or *sl* ^9^ and heterozygous for mutations under study by the absence of the marked balancer chromosomes. For rescue experiments, we likewise crossed males with the different rescue constructs to *sl^2^* mutant females, and selected male progeny hemizygous for *sl^2^* with the rescue construct.

### Body Weight and Length Measurements

For adult body weight measurements, sibling *sl^9^* homozygotes and *sl^9^/FM7* females were collected and aged for two days in groups of ten in fresh vials before use. Individual cold-killed flies were measured in a microbalance (Cahn C-31, USA) with 0.1 µg sensitivity and a range of 0.1 µg–25 mg. n = 70 flies/genotype. For pupal measurements, we crossed *sl^9^* males to *sl^9^/FM7* females in non-crowded conditions in fly bottles. Third instar larvae were recovered and sexed, and females, in groups of twenty, were housed in food vials. From these, female pupae from stages P12–P15 [43] were used. Pupae were weighed as above. Also, and similar to [15], [16], the long axis of the pupal case was measured for each pupae under a dissecting microscope with the aid of a micrometer grid.

### Analysis of wings

Flies were anesthetized with CO_2_ and wings were dissected, placed in absolute ethanol and mounted in a mixture of lactic acid:ethanol (6∶5) [10]. Wings were examined under a compound microscope. Measurements of wing surface and ectopic vein areas were made manually using IPLab software. For measurements of cellular density in wings, a small region between veins 3 and 4 was selected, and the total number of cells within this region was counted on one wing surface. In order to calculate cell densities, we divided the number of cells counted by the area surveyed (0.0298 mm^2^). This measurement was validated as representative of total wing area by counting the total number of cells in one wing surface in a few experiments.

### Scanning Electron Microscopy (SEM) of Whole Eyes

Flies were cold anesthetized, mounted and oriented sideways unto stubs by means of carbon paint. They were then viewed with a JEOL 1010 SEM under high vacuum conditions, with 20 KeV accelerating voltage, and whole eyes positioned perpendicular to the electron beam were digitized at 230x. Only one eye was accessible per fly. Digitized images were used to measure eye area and number of ommatidia using ImageJ.

### Eye Histology

Histology was performed according to Riesgo-Escovar, et al. [44]. Briefly, flies were anesthetized with CO_2_ and decapitated. Heads were placed on slides and cut in half by a medial sagittal section. The eyes were then fixed in 2% glutaraldehyde, 1% OsO_4_ in 1x cacodylate buffer (50 mM, pH 7.4) on ice for 30 minutes. The eyes were post-fixed in 2% OsO_4_ in 1x cacodylate buffer on ice for 2 hours. The OsO_4_ solution was then removed and the eyes were dehydrated through a graded acetone series. Absolute acetone was replaced with a mixture of Spurr's resin:acetone (1∶1) overnight. This mixture was replaced with 100% fresh Spurr's for at least 4 hours to allow the resin to infiltrate the tissue. The eyes were placed in plastic molds in 100% Spurr's and oriented using a fine needle. The molds were incubated at 60–65°C for 24–70 hours. Eyes were sectioned tangentially with a microtome (Leica RM2265) using glass knives. Sections of 1 µm thick were obtained, placed on slides, and stained with toluidine blue for approximately 30 seconds [45]. Finally, sections were mounted in Entellan (Electron Microscopy Sciences) and viewed on a compound microscope. Sections with fewer than 150 ommatidia were always selected for analysis, to assure that all ommatidia examined were at the level of the R7 rhabdomere.

### Analysis of loss-of-function clones in eyes

Patches of tissue lacking *sl* function were generated by the FLP-FRT system as described [46], by subjecting 2–4 day old larvae of genotype *y w sl* ^9^ FRT19A/FRT19A;hsFLP/+ to heat shock. Heads containing at least one *w* ^-^ clone were embedded in resin, sectioned and stained with toluidine blue as described [44], [45]. Ommatidia were scored for area of the R1–R6 rhabdomeres as a series of matched pairs from each head; each pair consisted of one *w+* ommatidium surrounded by all *w+* ommatidia, and one *w-* ommatidium surrounded by *w-* ommatidia. Each member of the pair was separated from the other member by no more than 3 other ommatidia, so that differences that occur due to the angle of section were minimized. The area of each rhabdomere was determined using the wand tool within ImageJ version 1.43u (Wayne Rasband, National Institutes of Health, Bethesda, MD).

### small wing constructs

A series of constructs were prepared from a 10 kb genomic DNA fragment (X10), that includes the entire *sl* transcription unit. The X10 fragment was subcloned into pBluescript KS, reisolated as a *Kpn*I/*Not*I digest and ligated into the *Kpn*I and *Not*I sites of *pCaSpeR*-4. Modified versions of X10 were produced by site-directed mutagenesis using the Quikchange kit (Stratagene). The X10 fragment was modified in R619 to A (R619 is a critical amino acid for the function of the N-SH2 domain) and independently in R726 to A (R726 is a critical amino acid for the function of the C-SH2 domain). The mutations were generated in a 1.7 kb *Bam*H1 fragment subcloned from X10 and confirmed by sequence analysis. The fragments were reconstructed in pBluescript and recloned into the *Kpn*I and *Not*I sites of *pCaSpeR*-4. The plasmids were injected into syncytial blastoderm embryos at a final concentration of 1 µg/ µl together with 0.1 µg/ µl Δ2–3 helper plasmid in a buffer containing 0.1 mM sodium phosphate (pH 7.8) and 5 mM KCl. The plasmids were detected by the presence of the *w* ^+^ gene marker of *pCaSpeR*-4 in transformed progeny [35].

### Statistical Analysis

Data are presented as mean ± SEM. The mean values were compared using Student's *t* test.

## Supporting Information

Figure S1 ***PKC53E*** **mutant flies have wing phenotypes.** Homozygous mutant *PKC53E^EY14093^* flies are viable and fertile, but have reduced wings (A) and ectopic wing veins (B), yet normal numbers of R7 photoreceptors in the eye (C). Each test is accompanied by corresponding control siblings. *p<0.001; error bars represent SEM, n = 100 for (A) and (B), and n = 4 eyes for each genotype, ≤150 ommatidia examined per eye.
(TIF)

Figure S2 **Reduced gene dosage of signaling genes on the** ***sl*** **^9^ ectopic wing vein phenotype.** n = 100. *p<0.001; error bars represent SEM.
(TIF)

Figure S3 ***aos*** **acts as a strong enhancer of** ***sl*** **in the eye.** (A) Shows a scanning EM of an eye from an *aos* heterozyote male fly, with normal morphology. (B) Shows a scanning EM of an eye from a male *sl^2^* fly, sibling to the fly in (C) that also carries a mutant copy of *aos* (*aos* ^Δ*7*^). Note slight roughness of the eye on (B), enhanced in the eye in (C). Scale bar in (B) is 100 micrometers.
(TIF)

Figure S4 **Reduced gene dosage of signaling genes on** ***sl*** **^1^ and** ***sl*** **^9^ extra R7 phenotypes.** (A, B) show histograms with the effects of heterozygosity for different signaling genes on the number of ommatidia with extra R7 cells in *sl* ^1^ (A) and *sl* ^9^ (B) mutants. n = 50–100 ommatidia per eye, from 4–7 eyes. *p<0.001; error bars represent SEM.
(TIF)

Figure S5 **Homozygosity for** ***sl*** **^2^ does not modify the dominant** ***S*** **^2^ R7 phenotype in the eye.** Histogram showing the average number of R7 cells per ommatidium in eyes from heterozygous *S* ^2^ flies, or from flies also mutant for *sl* ^2^. n = 5 eyes each with ≤150 ommatidia per eye. Error bars represent SEM.
(TIF)
