# Supplementary figures and images for: Insulin Receptor-Mediated Signaling via Phospholipase C-γ Regulates Growth and Differentiation in Drosophila
Source: PLoS One. 2011 Nov 21;6(11):e28067. doi: 10.1371/journal.pone.0028067 (PMC3221684; doi:10.1371/journal.pone.0028067)

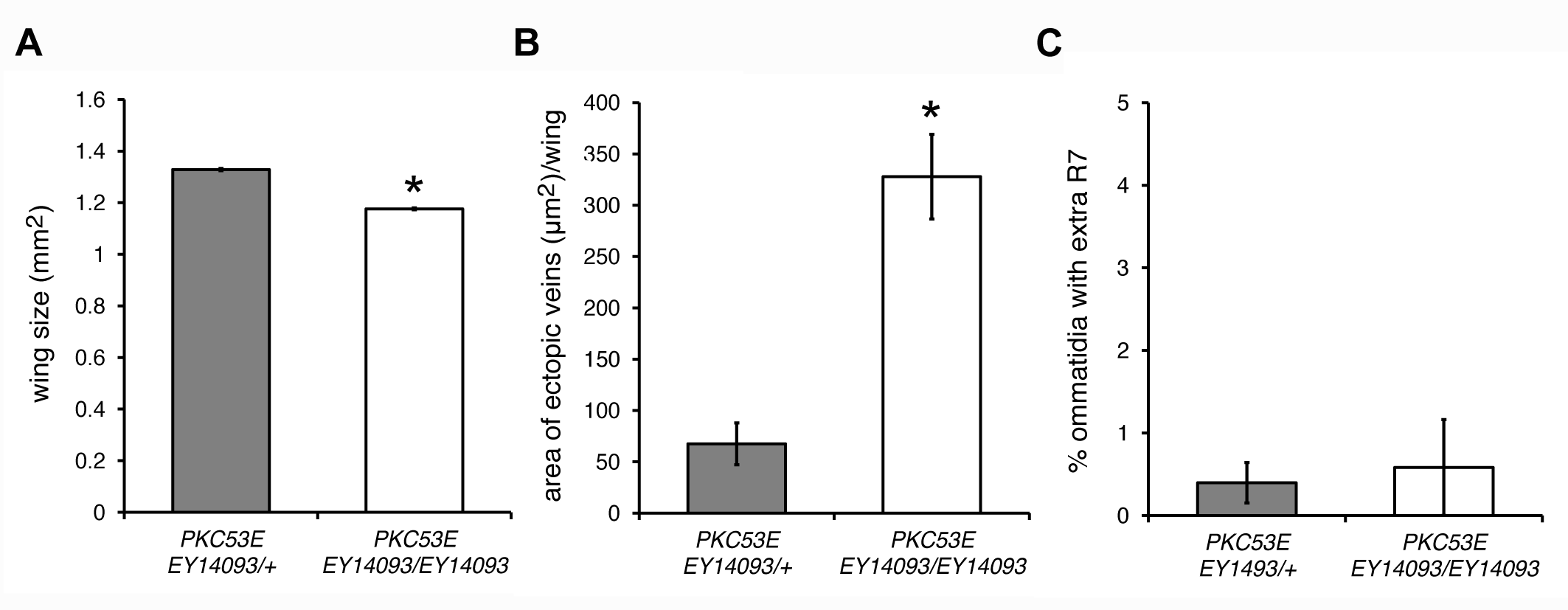

Supplement: Figure S1 — PKC53E mutant flies have wing phenotypes. Homozygous mutant PKC53EEY14093 flies are viable and fertile, but have reduced wings (A) and ectopic wing veins (B), yet normal numbers of R7 photoreceptors in the eye (C). Each test is accompanied by corresponding control siblings. *p<0.001; error bars represent SEM, n = 100 for (A) and (B), and n = 4 eyes for each genotype, ≤150 ommatidia examined per eye. (TIF) [file pone.0028067.s001.tif]

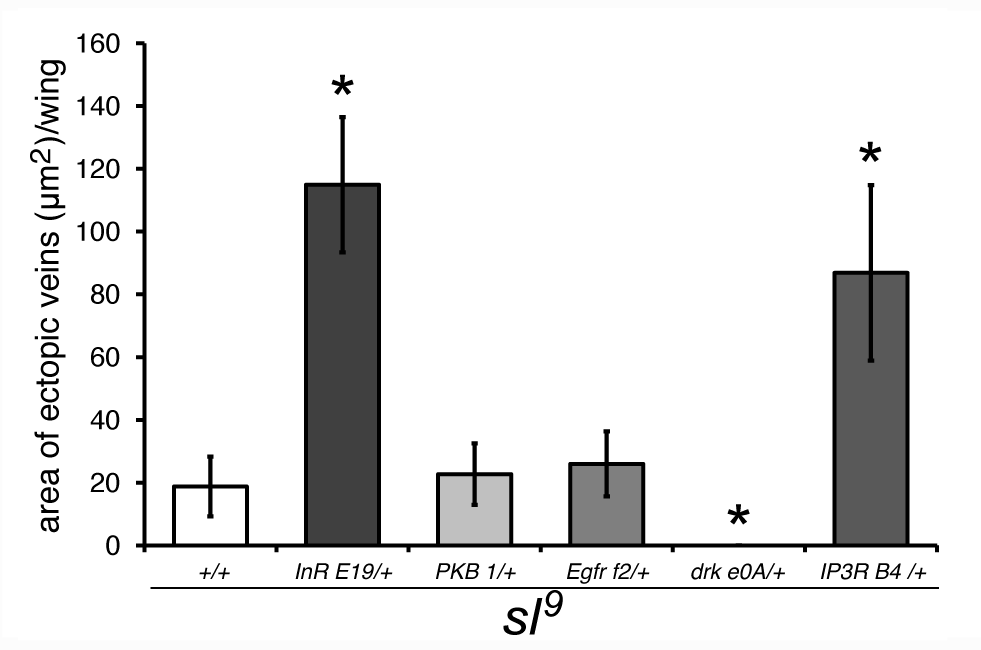

Supplement: Figure S2 — Reduced gene dosage of signaling genes on the sl 9 ectopic wing vein phenotype. n = 100. *p<0.001; error bars represent SEM. (TIF) [file pone.0028067.s002.tif]

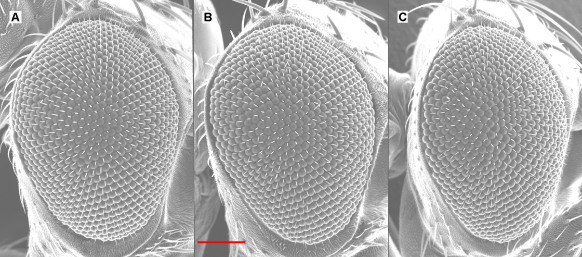

Supplement: Figure S3 — aos acts as a strong enhancer of sl in the eye. (A) Shows a scanning EM of an eye from an aos heterozyote male fly, with normal morphology. (B) Shows a scanning EM of an eye from a male sl2 fly, sibling to the fly in (C) that also carries a mutant copy of aos (aos Δ7). Note slight roughness of the eye on (B), enhanced in the eye in (C). Scale bar in (B) is 100 micrometers. (TIF) [file pone.0028067.s003.tif]

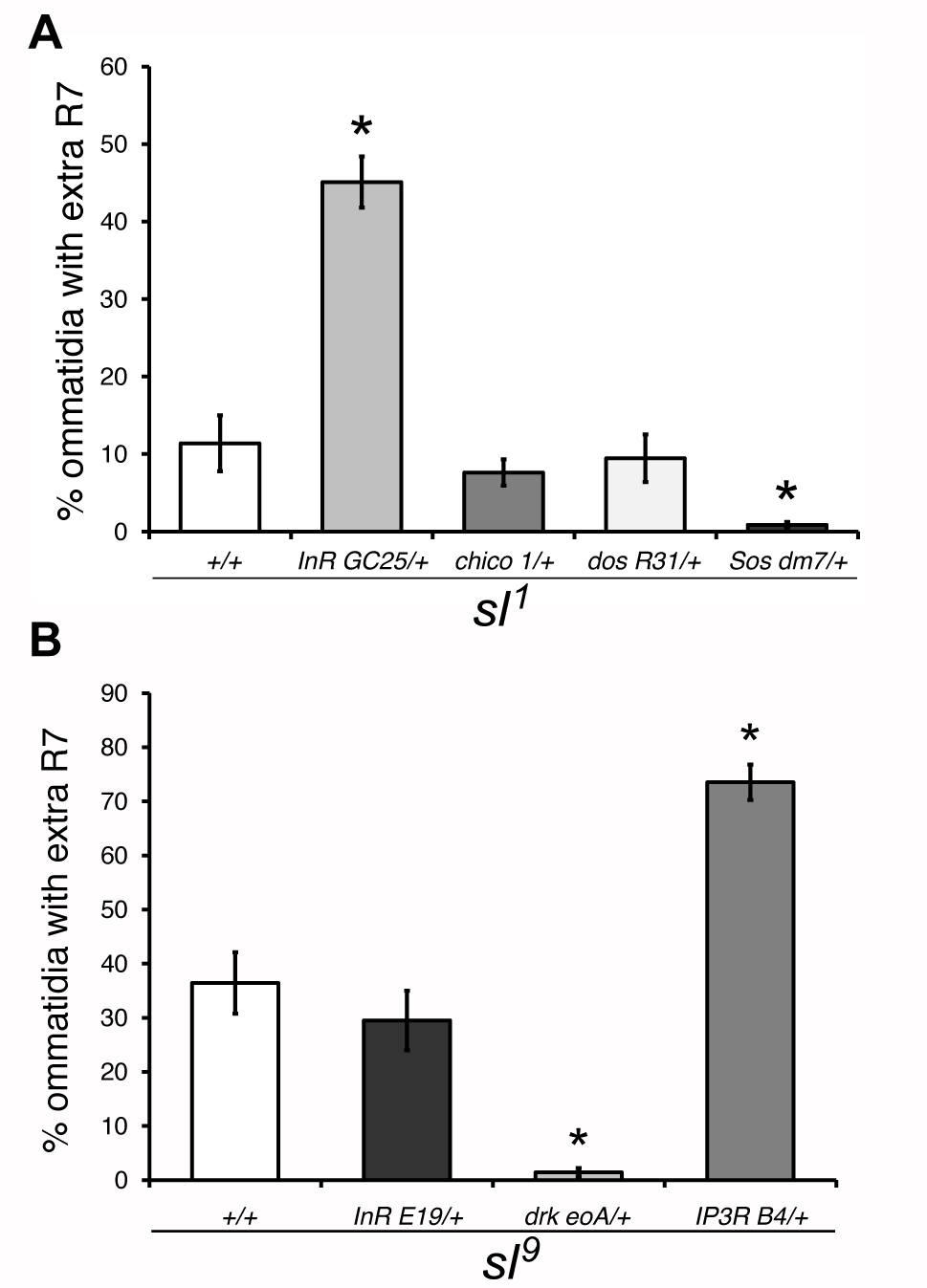

Supplement: Figure S4 — Reduced gene dosage of signaling genes on sl 1 and sl 9 extra R7 phenotypes. (A, B) show histograms with the effects of heterozygosity for different signaling genes on the number of ommatidia with extra R7 cells in sl 1 (A) and sl 9 (B) mutants. n = 50–100 ommatidia per eye, from 4–7 eyes. *p<0.001; error bars represent SEM. (TIF) [file pone.0028067.s004.tif]

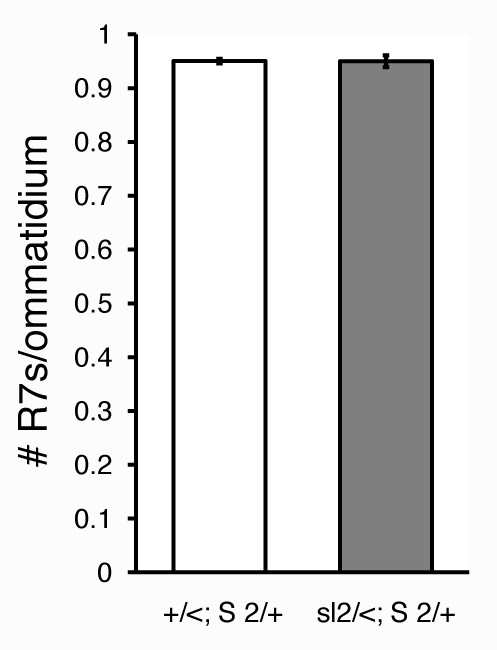

Supplement: Figure S5 — Homozygosity for sl 2 does not modify the dominant S 2 R7 phenotype in the eye. Histogram showing the average number of R7 cells per ommatidium in eyes from heterozygous S 2 flies, or from flies also mutant for sl 2. n = 5 eyes each with ≤150 ommatidia per eye. Error bars represent SEM. (TIF) [file pone.0028067.s005.tif]
